# Role of microRNA/lncRNA Intertwined With the Wnt/β-Catenin Axis in Regulating the Pathogenesis of Triple-Negative Breast Cancer

**DOI:** 10.3389/fphar.2022.814971

**Published:** 2022-06-24

**Authors:** Xue Hu, Qiang Zhang, Wanying Xing, Wan Wang

**Affiliations:** ^1^ Department of Breast Surgery, China-Japan Union Hospital of Jilin University, Changchun, China; ^2^ Department of Breast Surgery, Cancer Hospital of China Medical University, Liaoning Cancer Hospital & Institute, Shenyang, China

**Keywords:** breast cancer, lncRNA, microRNA, wnt/β-catenin, pathogenesis

## Abstract

**Objective (s):** In this mini-review, we aimed to discuss the Wnt/β-catenin signaling pathway modulation in triple-negative breast cancer, particularly the contribution of lncRNAs and miRNAs in its regulation and their possible entwining role in breast cancer pathogenesis, proliferation, migration, or malignancy.

**Background:** Malignant tumor formation is very high for breast cancer in women and is a leading cause of death all over the globe. Among breast cancer subtypes, triple-negative breast cancer is rife in premenopausal women, most invasive, and prone to metastasis. Complex pathways are involved in this cancer’s pathogenesis, advancement, and malignancy, including the Wnt/β-catenin signaling pathway. This pathway is conserved among vertebrates and is necessary for sustaining cell homeostasis. It is regulated by several elements such as transcription factors, enhancers, non-coding RNAs (lncRNAs and miRNAs), etc.

**Methods:** We evaluated lncRNAs and miRNAs differentially expressed in triple-negative breast cancer (TNBC) from the cDNA microarray data set literature survey. Using *in silico* analyses combined with a review of the current literature, we anticipated identifying lncRNAs and miRNAs that might modulate the Wnt/β-catenin signaling pathway.

**Result:** The miRNAs and lncRNAs specific to triple-negative breast cancer have been identified based on literature and database searches. Tumorigenesis, metastasis, and EMT were all given special attention. Apart from cross-talk being essential for TNBC tumorigenesis and treatment outcomes, our results indicated eight upregulated and seven downregulated miRNAs and 19 upregulated and three downregulated lncRNAs that can be used as predictive or diagnostic markers. This consolidated information could be useful in the clinic and provide a combined literature resource for TNBC researchers working on the Wnt/β-catenin miRNA/lncRNA axis.

**Conclusion:** In conclusion, because the Wnt pathway and miRNAs/lncRNAs can modulate TNBC, their intertwinement results in a cascade of complex reactions that affect TNBC and related processes. Their function in TNBC pathogenesis has been highlighted in molecular processes underlying the disease progression.

## Introduction

Breast cancer represents the most common type of cancer worldwide, with high morbidity and mortality rates, (Agustsson et al., 2020). Breast cancer shows high heterogeneity, which impacts the clinical course of the disease. Differential expression profiles among patients lead to tumor tissue heterogeneity, resulting in variations in malignant behavior, prognosis, and responsiveness to standard therapies (Padmanaban et al., 2019). This cancer is prevalent in women and is the first leading cause of cancerous death in women. Breast cancer has been progressively increasing in most nations (Ferlay et al., 2015). According to WHO data (https://www.who.int/news-room/fact-sheets/detail/breast-cancer), 2.3 million women were diagnosed with breast cancer in 2020, with about 685,000 fatalities. This year, in the United States alone, around 281,550 cases of invasive and 49,290 cases of non-invasive breast cancer cases were diagnosed in women, according to the American Cancer Society. Most deaths occur due to metastasis instead of the primary tumor in breast cancer (Geng et al., 2014). Metastasis is linked with stem cells, which have characteristics such as self-renewal, differentiation ability, drug resistance, etc. (Phi et al., 2018). These properties favor aggressive behavior in breast cancer stem cells, leading to recurrent and aggressive tumors on and away from the primary site. Upregulation of Wnt/β-catenin signaling has been observed in triple-negative breast cancer (TNBC)/basal-like cancer when collated with other breast cancer subtypes (luminal A, B, and HER2 positive) or normal tissues (Gangrade et al., 2018).

TNBC can be classified into at least six distinct subtypes with differences in clinical behavior and treatment response. TNBC is highly invasive and, due to the non-expression of estrogen receptor, progesterone receptor, or HER-2, it has poor prognosis, high metastatic potential, and is disposed to relapse (Yin et al., 2020). TNBCs are more common in younger and obese women, with premenopausal African American women having the highest prevalence. BRCA1 and BRCA2 gene mutations are identified in approximately 20% of TNBC patients. P53 and Rb1 mutations are also quite common in TNBC tumors (van Barele et al., 2021). Differentially expressed ncRNAs have been found in a variety of human cancers, including breast cancer. Several lncRNA molecules have been linked to tumorigenesis, and their differential expression could constitute a potential new category of biomarkers. The lncRNA HOTAIR (HOX transcript antisense intergenic RNA) was associated with the polycomb repressive complex 2 (PRC2) and the histone demethylation enzyme LSD1 (lysine-specific demethylase 1) in cancer cells, resulting in epigenetic changes that promote tumor development and metastasis (Rodrigues de Bastos and Nagai, 2020). Other circulating lncRNAs, including MALAT1, GAS5, H19, and MEG3, have also been linked to survival and treatment response. LncRNAs have opened up a new field of study for researchers all over the world, and these molecules have been assigned major roles that may have a direct impact on patient survival and therapeutic responsiveness (Gupta et al., 2010). The SPARC gene (secreted protein acidic and rich in cysteine, also known as osteonectin or basement-membrane protein 40) encodes a 32-kDa matricellular glycoprotein involved in a variety of biological activities, including differentiation, proliferation, migration, and adhesion (Zhang et al., 2019).

**FIGURE 1:**
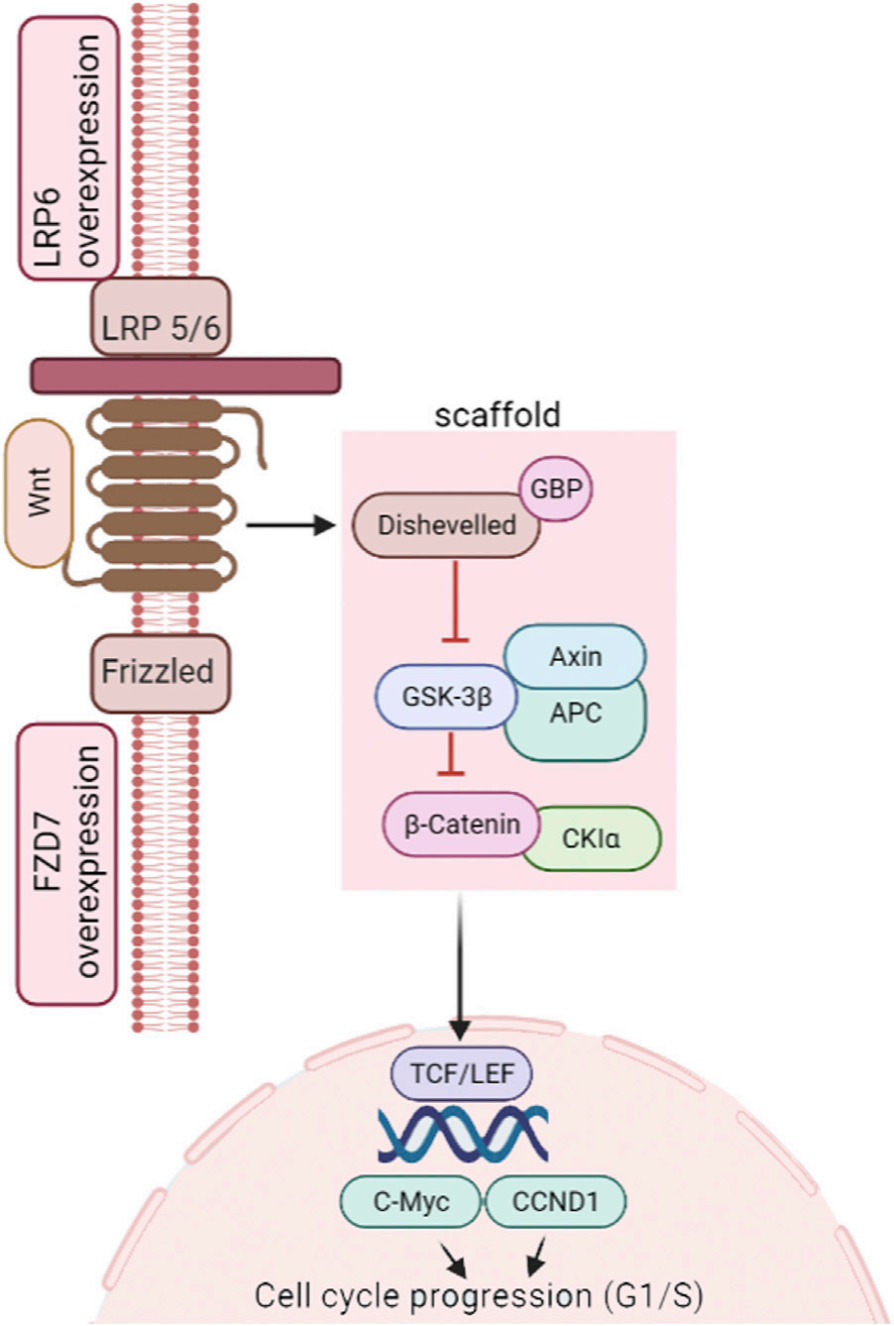
Wnt/β-catenin pathway in TNBC instigation. Figure adapted from the KEGG pathway (https://www.genome.jp/pathway/map05224).

The canonical Wnt/β-catenin signaling pathway contributes to instigation (Figure 1), differentiation, and proliferation of TNBC cells (Zhang et al., 2018), leading to primary tumor formation (Xu et al., 2015), cellular transition of the epithelial-to-mesenchymal (EMT) state, and metastasis (Pohl et al., 2017). Chemoresistance has also been linked with Wnt/β-catenin signaling, with impaired pathways leading to drug-resistant TNBC (Merikhian et al., 2021). This is due to the synergistic contact between the Wnt target gene c-MYC and HIF-1α. This dual gene interaction diminishes cancer cell response to the given drugs. However, knockdown of β-catenin has been reported to cause TNBC cells to respond to doxorubicin or cisplatin (Xu et al., 2015).

This is why the study of this pathway is essential in TNBC, and a comprehensive updated literature review was undertaken to integrate information regarding the Wnt/β-catenin signaling pathway and TNBC.

## Wnt/β-Catenin Signaling in TNBC

The Wnt pathway is tangled with low-density lipoprotein receptor-related protein5/6 (LRP5/6) and frizzled (FZD) receptors for its activation (King et al., 2012). If Wnts are insufficient or non-functional, β-catenin pools with a tetrad of proteins (axin, casein kinase 1 (CK1), adenomatous polyposis *coli* (APC) tumor suppressor, and glycogen synthase kinase-3b (GSK3b). Phosphorylation (by CK1 and GSK3b) is followed by ubiquitination and ultimate degradation of β-catenin (26S proteasome). Conversely, in the presence of the Wnt signal, β-catenin attaches to FZD and LRP5/6 receptors, leading to inhibition of GSK3b and stabilization of cytosolic β-catenin. This β-catenin is then shifted to the nucleus. It associates with T-cell factor/lymphoid-enhancing factor (TCF/LEF) to incite the downstream expression of cell development and cell cycle control genes (MacDonald et al., 2009). Secreted proteins regulate this signaling at the cell surface, where the central modulators are Wnt and R-spondin (Rspo). Inhibitors include Wnt Inhibitory Factor 1 (WIF1), Dickkopf (Dkk), soluble Frizzled-related protein (sFRP), and sclerostin (SOST) (Yao et al., 2011; Danieau et al., 2019). Abnormal Wnt signaling has been implicated in TNBC tumorigenesis (Xu et al., 2015; Mohammadi Yeganeh et al., 2017), stemness, metastasis, and prognosis (Ryu et al., 2020). Dey et al. (2013) identified that patients with dysregulated Wnt/β-catenin signaling had a higher chance of lung and brain metastases. Dysregulation of the canonical pathway is responsible for metastasis in more than half of breast cancer patients as the nucleolar β-catenin level is elevated. However, mutations in the relevant genes are uncommon (Yu et al., 2019). It implies that the role of the β-catenin is indispensable to the Wnt signaling in TNBC advancement, with the dissemination of non-phosphorylated cytoplasmic β-catenin to the nucleus having an imperative role in TNBC metastasis (Breuer et al., 2019; Satriyo et al., 2019).

Green et al. (2013) discovered that Wnt ligands caused enhanced transcription in the majority of TNBC cell lines, while Xu et al. (2015) reported that nuclear accumulation of β-catenin is linked with TNBC characteristics. The role of the key modulators of this pathway in TNBC includes the action of FZD receptors, LRP5/6, Receptor Tyrosine Kinase–Like Orphan Receptors (RORs), and Dead Box Proteins (DDX3 and DDX5) (Pohl et al., 2017). LRP5/6 is crucial in mammary development and is allied with tumorigenesis (Goel et al., 2012). Overexpression of LRP5/6 has been seen in TNBC (Maubant et al., 2018). LRP6 has also been linked with migration and invasion of cells (Ma et al., 2017). Elevated FZD7 expression in TNBC also promotes tumorigenesis (Yang et al., 2011) *via* transformation-related protein 63 (p63) (Chakrabarti et al., 2014), while FZD8-driven Wnt signaling mediated by c-Myc overexpression drives chemoresistance (Yin et al., 2013). Dead box proteins DDX3 and DDX5 have shown increased EMT in TNBC. DDX3 is associated with increased motility and invasiveness. In comparison, DDX5 is linked with tumorigenesis and cancer cell progression (Moore et al., 2010; Wang et al., 2012; Guturi et al., 2014; Pohl et al., 2017).

### Wnt/β-Catenin Signaling in TNBC Stem Cells

Cancer stem cells (CSCs) or cancer stem-like cells within the tumor, being less responsive to environmental stop signals, are responsible for cancer progression, metastasis, chemoresistance, and hence, cancer relapse (Clarke, 2019). They differ from other cancer cells as they use mitochondrial reactive oxygen species (ROS) for respiration, which means higher oxygen consumption, increased mitochondrial mass, and high resistance to DNA damage (Peiris-Pagès et al., 2018; Scatena et al., 2018). Abnormal Wnt/β-catenin signaling is linked with CSC formation and, hence, tumorigenesis, stemness, migration, and chemoresistance in TNBC (Alraouji et al., 2020). Upregulation of β-catenin triggers the CSC phenotype of TNBC, and repression by cadherin leads to suppression of this phenotype (Satriyo et al., 2019). Jang et al. (2015) observed that Wnt/β-catenin pathway genes (WNT1, FZD1, TCF4, and LEF1) are upregulated in CSC-enriched mammospheres in breast cancer, while signaling proteins (LEF1, TCF4, and β-catenin) were increased in high CSC activity, depicting cell fraction, compared to that with low CSC activity. However, recently, Brilliant et al. (2019) reported 11% of Wnt signaling expression in high vs. 33% in the low content of cancer stem cells. In contrast, some researchers have reported inhibiting TNBC CSCs by drugs targeting the Wnt/β-catenin pathway **(**like hydroxytyrosol) (Cruz-Lozano et al., 2019). Others have reported that β-catenin is also responsible for drug resistance (e.g., doxorubicin resistance) in TNBC CSCs (Xu et al., 2015).

### lncRNA and miRNAs Entangled With the Wnt/β-Catenin Pathway in TNBC

According to recent research, more than 90% of the transcripts in the human genome may not be able to code for proteins (Wilusz et al., 2009) but regulate the expression of nearby genes (He and Hannon, 2004; Trzybulska et al., 2018). They are categorized according to their size and function (Trzybulska et al., 2018), with microRNAs (miRNAs) being 19–24 nucleotides long and long non-coding RNAs (lncRNAs) being >200 nucleotides in length. There are other non-coding RNAs such as piwiRNAs, free circulating RNAs, and snoRNAs, but our focus will be on miRNAs and lncRNAs in this review. Long non-coding RNAs are abundant in human cells and play critical roles in a range of biological processes, including cell cycle regulation (Lu et al., 2016), genomic expression (Ballantyne et al., 2016), and cell differentiation (Chen et al., 2016). Increasing evidence has recently indicated that abnormal lncRNA expression is linked with various tumor forms (Mendell, 2016). Researchers have proved that miRNA dysregulation also leads to human cancer *via* different mechanisms (Karimzadeh et al., 2021), including altered epigenetics (Arif et al., 2020) and abnormal transcriptional control (Müller et al., 2019; Ali Syeda et al., 2020). Apart from performing as oncogenes, where they support proliferative signaling (Miao et al., 2017), invasion (Chen et al., 2017), repelling cell death, eluding progression suppressors, and metastasis, they act as tumor suppressors too (Hong et al., 2019). This has led to their demarcation as potential biomarkers of cancer (Gai et al., 2018; Dai et al., 2019).

miRNA expression in BCSCs and cancer cells signals that they are crucial for promoting characteristics such as stemness and tumorigenesis. Piasecka et al. (2018) reviewed 121 articles demonstrating the role of miRNAs in TNBC. After scanning a plethora of literature, it was revealed that the miRNAs not only serve as predictive markers of TNBC but also have prognostic clinical utility. They assist in attaining CSC properties in TNBC and EMT. Since these properties are conditions for metastasis, miRNAs play an essential role in cell transition to the metastasis stage. Several miRNAs, which are differently expressed in TNBC cells compared to normal cells and entangled with the Wnt/β-catenin pathway, have also been identified (Avery-Kiejda et al., 2014; Goh et al., 2016; Pohl et al., 2017).

More than 70 miRNAs (previously implicated in BC) have shown differential expression in TNBC, targeting 16 genes from the Wnt pathway, causing their increased expression in TNBC metastasis. The impacting miRNAs comprised the miR-17-92 oncogenic cluster members and the miR-200 family, revealing that most miRNAs are not mainly associated with a cancer subtype (Pohl et al., 2017). miR-340 alters CTNNB1, MYC, and ROCK1 gene expression of the Wnt pathway and causes apoptosis in TNBC (Mohammadi Yeganeh et al., 2017). miR-203 expresses higher methylation and is downregulated in TNBC, along with downregulation of the Wnt pathway (Taube et al., 2013). Telonis and Rigoutsos (2018) have identified miR-200c, miR-21, miR-17/92 cluster, and the miR-183/96/182 cluster to be upregulated in TNBC. Wang et al. (2019) demonstrated that miR-125, MiR, MiR-145, MiR-381, MiR-136, and MiR-4324 are associated with the poorest prognosis in TNBC patients. Thus, miRNAs play a critical role in TNBC and are intertwined with the Wnt pathway gene regulation, making them essential players in Wnt-mediated TNBC progression, prognosis, and other outcomes. Previously reported miRNAs with a role in TNBC regulation have been collected in Table 1 below:

**TABLE 1:** miRNAs impacting TNBC *via* the Wnt/β-Catenin axis.

S. No.	MiRNA	Upregulation or downregulation	Cell line	References
1	miR-142	Upregulated	HEK293T, MCF7, and MDA-MB-231	Isobe et al. (2014)
2	miR221/222	Upregulated	MDA-MB-231, MCF7, MDA-MB-468, Hs 578T, HCC1937, and MDA-MB-231, SKBR3, T47D, BT-474, 4T1	Liu et al. (2018)
3	miR-124	Upregulated	BT20, HCC70, 293T	Yang et al. (2020)
4	miR-125b	Upregulated	MDA-MB-468, MDA-MB-231, MCF-10A, and MCF-7	Nie et al. (2019)
5	miR-137	Downregulated	HCC38, MDA-MB-231, and MDA-MB-468	Cheng et al. (2019)
6	miR-29b-1	Downregulated	MB-231, MDA-MB-468, BT20, and HCC-1395	Drago-Ferrante et al. (2017)
7	miR-105	Upregulated	MB-361, MCF-7, BT-483, AU565, SkBR3, MCF-10A, MB-231 (MDA-MB-231), Hs578T, HCC1599, HCC1806, HCC1937, BT-549, DU4475, and HCC70	Li et al. (2017)
8	miR-93	Upregulated	MB-361, MCF-7, BT-483, AU565, SkBR3, MCF-10A, MB-231 (MDA-MB-231), Hs578T, HCC1599, HCC1806, HCC1937, BT-549, DU4475, and HCC70	Li et al. (2017)
9	miR-27a	Upregulated	BT-549, MDA-MB-231, MDA-MB-468, MDA-MB-453, MCF-10A, and DU4475	Wu et al. (2020)
10	miR-130a	Downregulated	MDA-MB-468, MDA-MB-231, and MF-10A	Poodineh et al. (2020)
11	miR-384	Downregulated	MCF-7; MDA-MB-231	Wang et al. (2018a)
12	miR-34a	Downregulated	SUM159PT, mammospheres and Comma-Dβ cells	Bonetti et al. (2019)
13	miR-374a	Downregulated	MCF7, T47D, BT474, MDA-MB-231, MDA-MB-435, MDA-MB-468, and 4T1	Cai et al. (2013)
14	miR-340	Downregulated	MCF-10A, MDA-MB-231, and HEK 293T	Mohammadi Yeganeh et al. (2017)
15	miR-218	Upregulated	MCF-10A, MCF-7, and MDA-MB-231	Taipaleenmäki et al. (2016)

**FIGURE 2:**
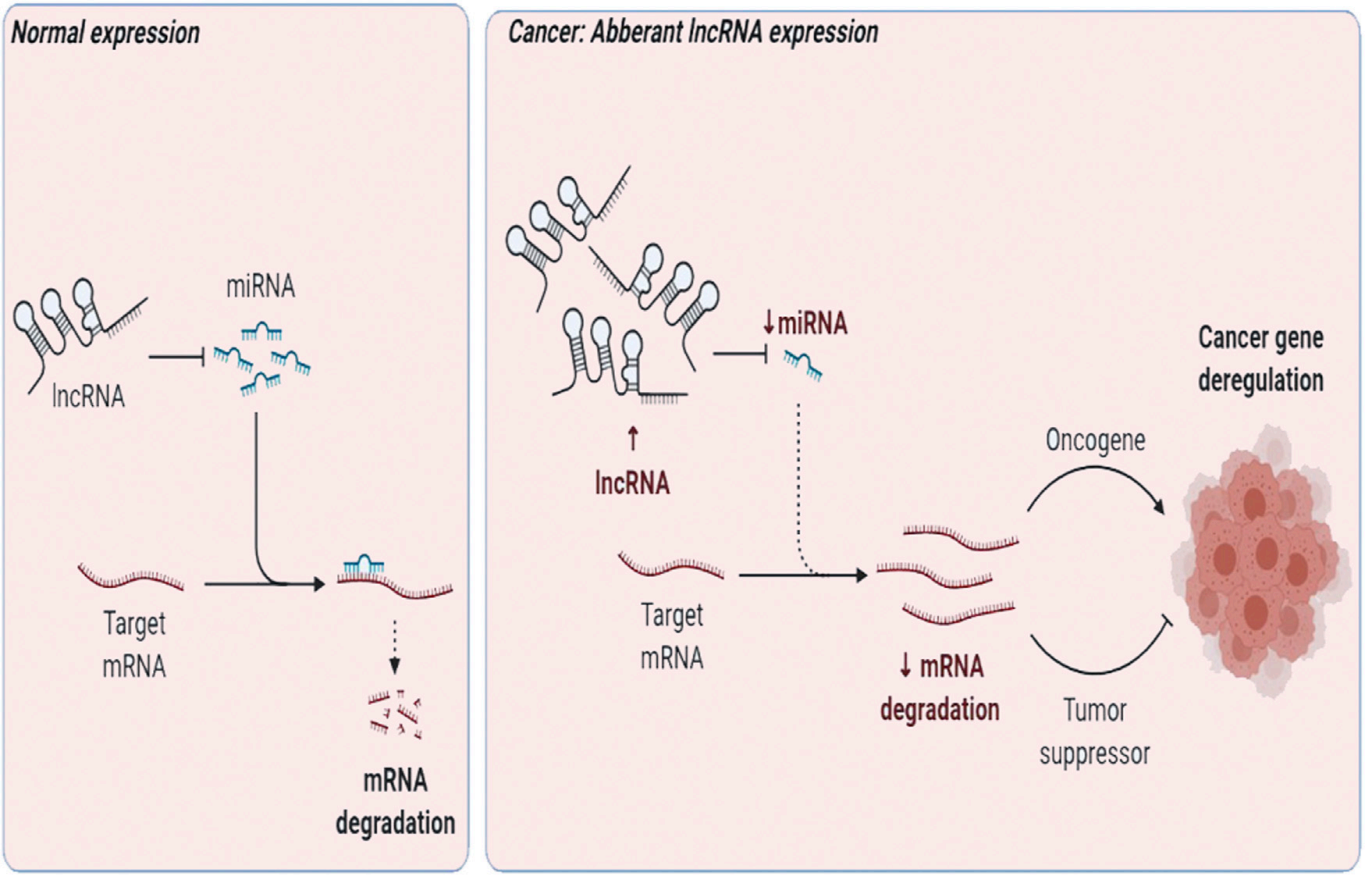
LncRNA–miRNA gene expression regulation in normal vs. cancer phenotype.

LncRNAs may support transcription; aid RNA interference; act as a decoy, peptide, and scaffold; or function as a guide/enhancer RNA (Li et al., 2014). They may deactivate miRNAs in cancer *via* the “sponge” effect, that is, act as a competing molecule or decoy to attach to miRNAs and perturb them from their target (Fan et al., 2018; Huang et al., 2019) (Figure 2). They have been demarcated as diagnostic and therapeutic targets. Here, we review lncRNA intertwined with the Wnt pathway and TNBC progression, pathogenesis, prognosis, or invasion. LncRNA Lung Cancer–Associated Transcript 1 (LUCAT1) is interlinked with miR-5582 and regulates breast cancer stemness *via* the Wnt/β-catenin pathway (Zheng et al., 2019). UCA1 promotes EMT (Xiao et al., 2016), while actin filament–associated protein 1 antisense RNA1 (AFAP1-AS1) promotes EMT and tumorigenesis (Zhang et al., 2018). and differentiation antagonizing non-protein coding RNA (DANCR) negatively regulates the Wnt pathway (Li and Zhou, 2018) to uplift tumorigenesis (Tao et al., 2019), EMT, and stemness in TNBC (Zhang and Wang, 2020). HOX Antisense Intergenic RNA (HOTAIR) modulates the Wnt pathway (Li et al., 2016) and leads to metastasis (Collina et al., 2019) and poor prognosis in TNBC *via* upregulation by miR-146a (Liang et al., 2019).

Jiang et al. (2020) reported that DiGeorge Syndrome Critical Region Gene 5 (DGCR5) induces tumorigenesis in TNBC. LncRNA associated with poor prognosis of hepatocellular carcinoma (AWPPH) promotes TNBC growth by upregulating the frizzled homolog 7 (FZD7) ligand of the Wnt pathway (Wang et al., 2018b) and decreased manifestation of lncRNA has been reported to increase the malignant spread of TNBCs (Liu et al., 2017). Long intergenic non-protein coding RNA 1234 (LINC01234) modulates TNBC cell growth, invasion, and EMT positively (Xiao et al., 2021). We also mined lncRNAs with a role in TNBC and impacted by Wnt pathway genes (Table 2) from the lnc2Cancer 3.0 database (Gao et al., 2021).

**TABLE 2:** LncRNAs impacting TNBC *via* the Wnt/β-Catenin axis mined from the lnc2 Cancer database.

S. No.	Name	Method of identification	Expression pattern	References
1	ANRIL	qPCR, luciferase reporter assay, RIP.	Upregulated	Xu et al. (2017)
2	AFAP1-AS1	qPCR, Western blot, *in vitro* knockdown etc.	Upregulated	Zhang et al. (2018)
3	AWPPH	qRT-PCR etc.	Upregulated	Liu et al. (2019)
4	CCAT1	qPCR, luciferase reporter assay, Western blot	Upregulated	Han et al. (2019)
5	DANCR	qPCR, Western blot, *in vitro* knockdown, RIP etc.	Upregulated	Tang et al. (2018a)
6	FAM83H-AS1	qRT-PCR, Western blot	Upregulated	Han et al. (2020)
7	GAS5	qPCR, Western blot, Luciferase reporter assay	Downregulated	Li et al. (2018)
8	H19	qPCR, Western blot, other	Upregulated	Han et al. (2018)
9	HOTAIR	Microarray, qPCR etc.	Upregulated	Chen et al. (2015)
10	LINC00052	Microarray, qPCR etc.	Downregulated	Lv et al. (2016)
11	LINC00115	qRT-PCR, Western blot	Upregulated	Yuan et al. (2020)
12	LINC00152	qPCR, Western blot	Upregulated	Wu et al. (2018)
13	LINC00173	qPCR	Upregulated	Fan et al. (2020)
14	LINC01133	qPCR, Western blot	Upregulated	Tu et al. (2019)
15	LUCAT1	qPCR, Western blot, a luciferase reporter assay, *in vitro* knockdown, RIP	Upregulated	Mou and Wang, (2019)
16	MALAT1	Microarray, qPCR etc.	Upregulated	Chen et al. (2015)
17	NEAT1	qPCR, Western blot	Upregulated	Shin et al. (2019)
18	PCAT6	qPCR, Western blot, luciferase reporter assay, *in vitro* knockdown etc.	Upregulated	Shi et al. (2020)
19	PVT1	qPCR, Western blot, RNAi, other	Upregulated	Wang et al. (2018c)
20	SOX21-AS1	qPCR, Western blot, luciferase reporter assay etc	Upregulated	Liu et al. (2020)
21	XIST	qRT-PCR, Western blot	Downregulated	Li et al. (2020)
22	ZEB1-AS1	qRT-PCR, RIP, dual luciferase reporter assay	Upregulated	Luo et al. (2020)

In addition, lncRNA–miRNA interactions entwining the Wnt pathway have also been noted in TNBC (Volovat et al., 2020). Among these, intranuclear Metastasis-Related Lung Adenocarcinoma Transcript 1 (MALAT1) acts as a sponge of miR-129-5p (Dong et al., 2015), and silencing of this non-coding gene causes a decline in cell propagation and movement, illustrating its role in TNBC pathology (Zuo et al., 2017). AWPPH stimulates cell proliferation and contributes to drug therapy resistance when combined with miRNA-21 and is being exploited as a diagnostic biomarker (Cascione et al., 2013; Liu et al., 2019). At the same time, TUG1 impacts miR-197, prompts NLK expression, and incapacitates the Wnt signaling pathway, making the TNBC cells susceptible to cisplatin therapy (Tang et al., 2018b). AFAP1-AS1 controls miRNA-2110, leading to tumorigenesis and cell invasion (Zuo et al., 2017). The diminished NEF and boosted miRNA-155 levels segregate TNBC patients from controls, suggesting an interlinked modulation prompting enhanced invasion and cell migration in the case of increased miRNA-155 (Song et al., 2019).

## Conclusion

This narrative review was centered on TNBC association with the Wnt/β-catenin pathway. Moreover, miRNAs and lncRNAs shown to be specific to triple-negative breast carcinoma were listed, both from literature and database searches. Since the Wnt pathway and miRNAs/lncRNAs can modulate TNBC and their intertwinement forms a cascade of complex reactions that impact TNBC and associated processes, their role in TNBC pathology was highlighted concerning molecular processes underlying disease progression. Particular emphasis was put on tumorigenesis, metastasis, and EMT. Apart from cross-talk critical for TNBC tumorigenesis and treatment outcomes, miRNA and lncRNA can serve as predictive or diagnostic markers, so this consolidated information might be of clinical use and offer a consolidated literature resource to scientists working on the Wnt/β-catenin and miRNA/lncRNA axis of TNBC. They also have a role in chemotherapy resistance. However, information on detailed analyses of Wnt/miRNA/lncRNA axis mechanisms in TNBC is still scant, and more work needs to be carried out to infer the pivotal role of these moieties in TNBC. Owing to the heterogeneity of TNBC, recognition of subgroups or their pathologies based on the varied signatures of this axis could be interesting to explore. Furthermore, knock-out or knock-in functional studies in model organisms could be beneficial to understanding the comprehensive role of this axis in TNBC.
